# Primary extramedullary plasmacytoma of the nasal cavity: a case report of a resource-limited setting

**DOI:** 10.1097/RC9.0000000000000593

**Published:** 2026-06-18

**Authors:** Diwakar Koirala, Bivek Mishra, Ramesh Sapkota, Suman Supratik, Yamuna Agrawal, Shyam Thapa Chhetri

**Affiliations:** B.P. Koirala Institute of Health Sciences, Dharan, Nepal

**Keywords:** extramedullary plasmacytoma, maxillectomy, nasal cavity, plasma cell neoplasm

## Abstract

**Introduction and importance:**

Extramedullary plasmacytoma (EMP) is an uncommon plasma cell neoplasm, representing approximately 3% of all plasma cell disorders, with a predilection for the upper aerodigestive tract. Lesions involving the nasal cavity and paranasal sinuses are exceedingly rare and may mimic other sinonasal malignancies, posing diagnostic challenges in resource-limited settings.

**Case presentation:**

A 59-year-old female presented with a gradually progressive right nasal obstruction associated with occasional epistaxis and nasal discharge. Examination revealed a greyish-white irregular mass occupying the right nasal cavity. Computed tomography demonstrated a 6.2 × 3.7 × 6.5 cm mildly enhancing lesion within the right ethmoidal sinus, extending into adjacent structures, suggestive of malignancy. Histopathology confirmed plasmacytic proliferation consistent with EMP. Immunohistochemistry for S100, recommended to exclude melanoma, could not be performed due to resource constraints. The patient underwent wide local excision with right total maxillectomy under general anesthesia. Postoperative recovery was uneventful, and she was discharged after 7 days with advice for prosthetic rehabilitation.

**Clinical discussion:**

EMP is a radiosensitive tumor managed with surgery, radiotherapy, or a combination of both. Differentiation from multiple myeloma and other plasma cell neoplasms requires clinical, radiological, and pathological correlation. Regular follow-up is crucial to monitor recurrence or progression to myeloma.

**Conclusion:**

Sinonasal EMP is a rare but treatable entity. Accurate diagnosis, complete excision, and consistent follow-up are essential for achieving favorable outcomes, particularly in resource-constrained environments.

## Introduction

A plasmacytoma is an uncommon, well-defined solitary tumor composed of neoplastic monoclonal plasma cells, initially described by Schridde in 1905^[^1^]^. These lesions are categorized based on their site of origin as either arising from soft tissue or from the skeletal system. Extramedullary plasmacytomas (EMPs) constitute approximately 3% of all plasma cell neoplasms, with the majority (80%) arising in the head and neck region^[^2^]^. They account for about 4% of tumors occurring in the nasal cavity. Patients with solitary EMPs are characterized by biopsy-confirmed extramedullary tumors demonstrating clonal plasma cells, absence of clonal plasma cells in bone marrow aspirate and biopsy, no lytic lesions detected by bone survey, positron emission tomography/computed tomography (CT), or magnetic resonance imaging of the spine and pelvis, and no systemic manifestations of disease such as anemia or hypercalcemia^[^3^]^. Plasmacytomas can be classified into two^[^4^]^ clinical subtypes: solitary plasmacytoma of bone and extramedullary (or extraosseous) plasmacytoma. EMPs are uncommon, comprising approximately 1% of all plasma cell neoplasms. While they most frequently arise in the upper aerodigestive tract, they may also occur in other locations, including lymph nodes, bladder, breast, thyroid, testes, parotid glands, skin, and central nervous system^[^5^]^.

HIGHLIGHTS

- Rare nasal extramedullary plasmacytoma presenting as a sinonasal mass.
- Diagnostic challenge in resource-limited settings without full immunohistochemistry.
- Computed tomography showed a large, destructive ethmoidal mass mimicking malignancy.
- Managed successfully with wide excision and total maxillectomy.
- Emphasis on follow-up to detect recurrence or progression to myeloma.

The case has been written in accordance with the SCARE Criteria-2025^[^6^]^.

## Case presentation

A 59-year-old female was admitted to the Department of Otorhinolaryngology. She presented with a mass arising from the right nasal cavity. The patient had a gradual onset of nasal obstruction on the right side, associated with occasional epistaxis and nasal discharge for several months. There was no history of trauma, visual disturbances, or systemic symptoms.

**Figure 1.**
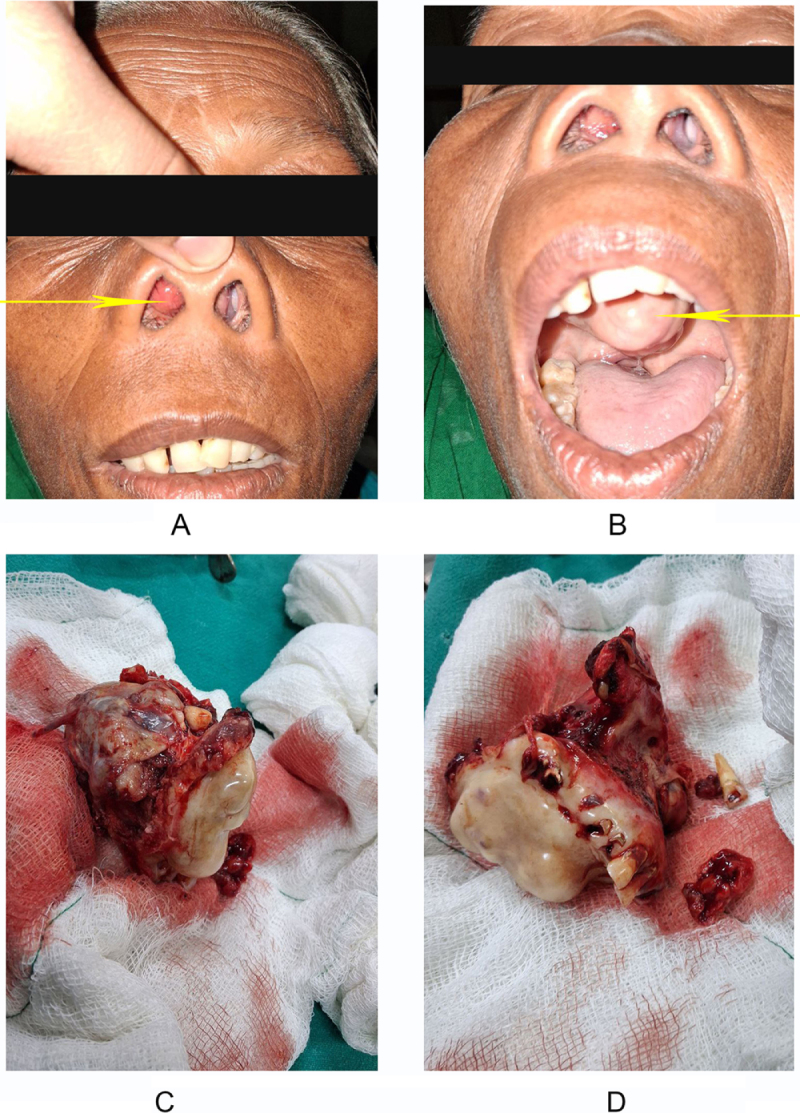
(A and B) Preoperative images with the extent of the mass. (C and D) Postoperative images of the excised mass.

On examination, the general condition of the patient was stable. Vital parameters were within normal limits. Local examination of the nose revealed a greyish-white irregular soft tissue mass occupying the right nasal cavity with partial obstruction. The septum appeared deviated to the left side, and the mass was seen extending toward the choana and possibly involving the ethmoidal region. There was no facial deformity, tenderness, or lymphadenopathy. Oral cavity and oropharyngeal examinations were unremarkable. The extent of the mass can be appreciated in Fig. 1 (A and B).

Investigations were performed during admission. Complete blood count showed hemoglobin 11.2 g/dL, total leucocyte count 14 200/mm^3^, and platelet count 3.98 lakhs/mm^3^. Differential leucocyte count showed neutrophils 72%, lymphocytes 24%, and eosinophils 4%. The elevated total leucocyte count with neutrophilic predominance likely reflects a reactive inflammatory response rather than a specific marker of malignancy. In resource-constrained settings, where advanced diagnostic modalities are unavailable, such basic laboratory parameters may provide supportive but nonspecific information and should be interpreted in conjunction with clinical and radiological findings. Coagulation profile was within normal limits. Blood sugar, renal function, and electrolytes were normal. Liver function tests revealed total bilirubin 0.45 mg/dL, direct bilirubin 0.12 mg/dL, alanine aminotransferase 10.36 U/L, aspartate aminotransferase 7.25 U/L, and total protein 6.55 g/dL. Serological tests for HIV, hepatitis b surface antigen, and hepatitis c virus were negative.

Due to resource limitations, advanced investigations such as serum protein electrophoresis, immunofixation, free light chain assay, urine Bence-Jones protein analysis, and bone marrow biopsy could not be performed. However, the patient demonstrated no clinical or laboratory features suggestive of systemic plasma cell myeloma, including the absence of anemia, hypercalcemia, renal dysfunction, or lytic bone lesions on imaging, supporting the diagnosis of a solitary EMP in the given clinical context.

**Figure 2.**
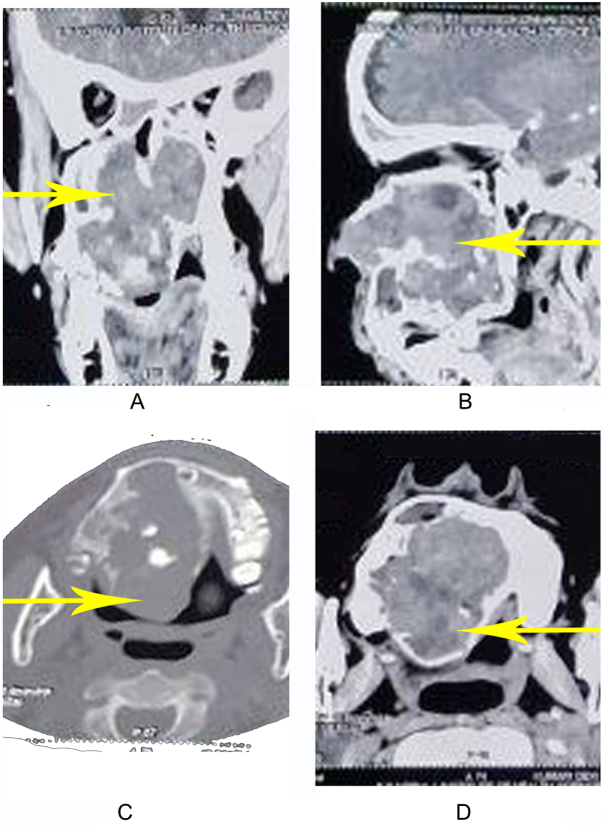
(A) Coronal slice of the extent of the lesion. (B) Sagittal slice of the extent of the lesion. (C and D) Transverse slices of the extent of the lesion.

CT scan of the nose and paranasal sinuses demonstrated a large, mildly enhancing mass measuring approximately 6.2 × 3.7 × 6.5 cm within the right ethmoidal sinus, extending into the nasal cavity and both frontal sinuses. There was bony erosion and destruction of the ethmoidal septa, extending into the left ethmoidal air cells and posteriorly toward the nasopharynx. The lesion extended inferiorly, with erosion of the alveolar process and partial destruction of the hard palate. No intracranial or intraorbital extension was seen. Visualized brain parenchyma and nasopharynx were normal. The CT images are shown in Fig. 2. The impression was of a likely sinonasal malignancy, for which histopathological correlation was advised.

An initial histopathology report of the nasal mass described multiple bits of greyish-white irregular soft tissue measuring 0.4 × 0.3 × 0.1 cm. Microscopy revealed stratified squamous and ciliated columnar epithelium exhibiting hyperplasia and dysplasia, with loss of polarity, increased nuclear-cytoplasmic ratio, coarse chromatin, and visible nucleoli. The subepithelium showed inflammatory infiltrates with lymphocytes and plasma cells. The features were consistent with high-grade dysplasia.

**Figure 3.**
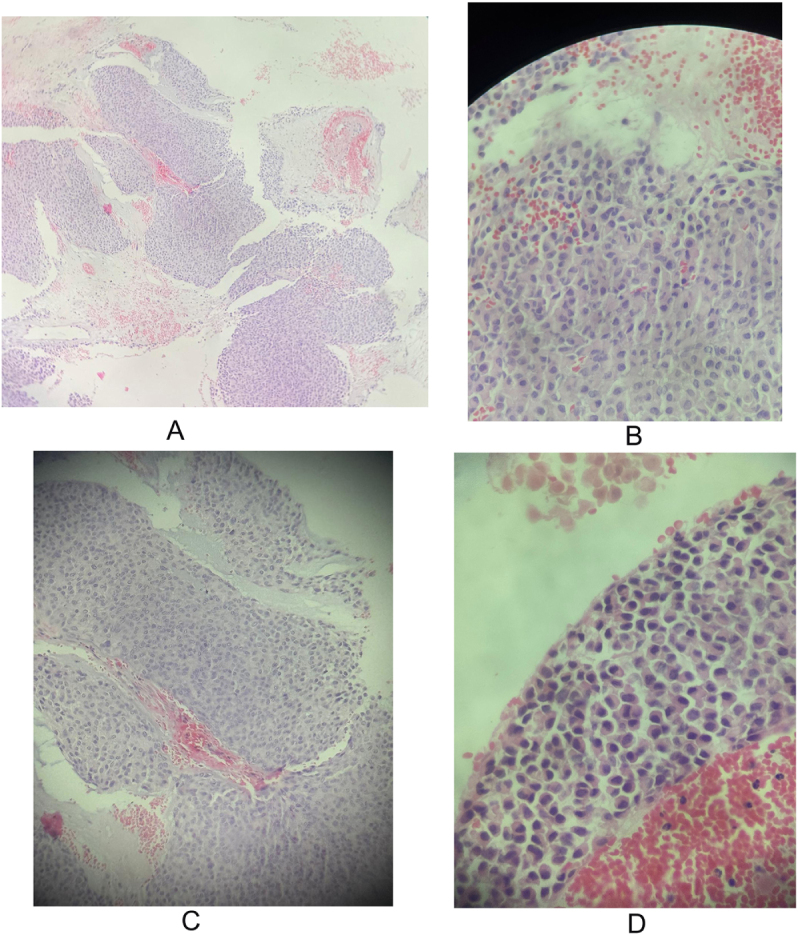
(A) (4×), (B and C) (10×) Low-power view shows tumor cells arranged in a papillary pattern with a fibrovascular core. (D) (40×): High-power view shows tumor cells having eccentrically placed nuclei and abundant eosinophilic cytoplasm, with a plasmacytoid appearance of the cells.

Subsequent repeat biopsy of the right nasal cavity mass was performed. The specimen, consisting of multiple bits of grey-white to grey-brown soft tissue measuring 6 × 4 × 3 cm, was examined histologically. Microscopy showed proliferation of plasma cells arranged in sheets and clusters with eccentric nuclei, coarse chromatin, and moderate cytoplasm. Occasional binucleate forms and mitotic figures were noted. Areas of hemorrhage and necrosis were present. The stroma revealed congested blood vessels, lymphocytes, and histiocytes, as shown in Figure 3.

The differential diagnoses considered in the pathology report were mucosal melanoma and EMP. In view of mucosal melanoma as a differential diagnosis, clinical and radiological assessment did not reveal regional lymphadenopathy or distant metastasis. Surgical excision was planned with adequate oncological margins, considering the possibility of malignancy. Regional lymph node dissection was not performed due to the absence of clinically or radiologically suspicious nodes. The decision to proceed with surgery in the absence of complete immunohistochemistry was based on the aggressive radiological features and the need for definitive management in a resource-limited setting. Immunohistochemical analysis for S100, which aids in differentiating melanoma from plasmacytoma, was recommended; however, due to constraints inherent to our resource-limited setting, the investigation could not be performed.

The patient was scheduled for wide local excision with right total maxillectomy under general anesthesia. The procedure was performed via a lateral rhinotomy approach, allowing adequate exposure of the tumor. Intraoperative hemostasis was achieved using electrocautery, and careful dissection was performed to preserve surrounding vital structures. No major vascular ligation, including branches of the internal maxillary artery, was required. The tumor was excised en bloc with adequate margins. No significant intraoperative complications were encountered. The procedure was uneventful, and the patient was discharged after a 7-day hospital stay with advice to obtain an obturator to address postoperative facial asymmetry. The excised mass is shown in Fig. 1(C and D).

## Discussion

In adults, EMPs are most commonly localized to the upper aerodigestive tract, although rare occurrences involving lymph nodes and the gastrointestinal tract have also been reported^[^7^]^. When evaluating a neoplastic lesion composed of a monoclonal population exhibiting distinct plasmacytic differentiation, the differential diagnoses should encompass plasma cell neoplasms – such as plasma cell myeloma and plasmacytoma – as well as B-cell lymphomas with plasmacytic features, including extranodal marginal zone lymphoma (MZL) and lymphoplasmacytic lymphoma (LPL). Plasma cell myeloma typically presents as a multifocal, bone marrow–based plasma cell proliferation associated with monoclonal protein in the serum or urine and often results in organ or tissue damage, none of which were observed in our patient. Although EMP (extraosseus plasmacytoma [EOPC]) and extramedullary myelomatous involvement cannot be reliably distinguished on morphologic grounds alone, the latter usually demonstrates CD56 expression and may show positivity for cyclin D1 and p53, whereas EOPC rarely expresses these markers^[^8^]^. EMP is recognized for its high radiosensitivity and is typically managed with radiotherapy, surgical excision, or a combination of both modalities. The overall outcomes associated with these treatment approaches are generally favorable^[^9^]^. However, our case only involved surgical resection without radiation therapy.

The definitive diagnosis of plasmacytoma relies on histopathological confirmation of a solitary plasma cell neoplasm, which may or may not be associated with a monoclonal gammopathy, in conjunction with the absence of plasma cell myeloma on bone marrow biopsy. Additionally, affected patients typically exhibit no clinical or laboratory evidence of hypercalcemia, anemia, or renal dysfunction^[^10^]^. The prognosis of EMP is generally favorable; however, local recurrence is observed in approximately 30% of cases, and systemic dissemination occurs in about 40%. Progression to multiple myeloma has been reported in 17–33% of patients, compared with solitary osseous plasmacytoma, which demonstrates a higher transformation rate exceeding 50%^[^11^]^.

Because EMPs and multiple myeloma exhibit similar tumor cell morphology and overlapping immunohistochemical profiles, their differentiation necessitates the integration of additional clinical, biochemical, and radiological findings. Differentiating plasmacytoma from multiple myeloma is clinically significant, as over 60% of patients with solitary plasmacytoma achieve a cure with local therapy alone, whereas the 5-year survival rate for plasma cell myeloma is approximately 35%. The most concerning complication remains progression to multiple myeloma^[^4^]^. Primary EMP may relapse through local recurrence, transformation into multiple myeloma, or distant metastasis. Most recurrences are localized and demonstrate a good response to radiotherapy. The combination of chemotherapy with radiotherapy is recommended to enhance local disease control and improve cure rates^[^12^]^.

In the present case, the patient underwent wide local excision with right total maxillectomy without adjuvant chemotherapy or radiotherapy. The decision to proceed with surgical excision was primarily guided by the extensive local disease, including bony destruction and involvement of adjacent sinonasal structures, which necessitated complete resection for local control. Additionally, limitations in access to radiotherapy facilities and the need for immediate symptom relief influenced the treatment approach. Postoperative radiotherapy was not administered due to these constraints and the absence of residual disease clinically. Postoperatively, the patient was placed under regular follow-up at 3-month intervals for surveillance of recurrence. The overall clinical outcome and prognosis were favorable.

## Conclusion

EMP of the nasal cavity is a rare neoplasm that can mimic other sinonasal malignancies, underscoring the importance of histopathological evaluation for definitive diagnosis. Surgical excision, with or without adjuvant radiotherapy, remains the cornerstone of management, yielding favorable outcomes in most cases. Regular postoperative surveillance is essential for early detection of recurrence or progression to multiple myeloma. This case highlights the significance of thorough clinicopathological correlation, appropriate imaging, and diligent follow-up in ensuring optimal prognosis, particularly in resource-limited settings where advanced immunohistochemical analysis may not always be feasible.

## Data Availability

Data not generated for case report.
